# Whey-Derived Porous Carbon Scaffolds for Bone Tissue Engineering

**DOI:** 10.3390/biomedicines9091091

**Published:** 2021-08-26

**Authors:** Raúl Llamas-Unzueta, Marta Suárez, Adolfo Fernández, Raquel Díaz, Miguel A. Montes-Morán, J. Angel Menéndez

**Affiliations:** 1 Instituto de Ciencia y Tecnología del Carbono (INCAR-CSIC), c/Francisco Pintado Fe, 26, 33011 Oviedo, Spain; r.llamas@incar.csic.es (R.L.-U.); miguel.montes@csic.es (M.A.M.-M.); 2 Nanomaterials and Nanotechnology Research Center (CINN-CSIC), Universidad de Oviedo (UO), Principado de Asturias, Avda de la Vega 4-6, 33940 El Entrego, Spain; m.suarez@cinn.es (M.S.); a.fernandez@cinn.es (A.F.); r.diaz@cinn.es (R.D.); 3 Instituto de Investigación Sanitaria del Principado de Asturias, Av. Roma, s/n, 33011 Oviedo, Spain

**Keywords:** whey, scaffolds, tissue engineering, porous carbon, biomaterials

## Abstract

Porous carbon structures derived from whey powders are described and evaluated as potential scaffolds in bone tissue engineering. These materials have a porosity between 48% and 58%, with a hierarchical pore size distribution ranging from 1 to 400 micrometres. Compressive strength and elastic modulus are outstanding for such a porous material, being up to three times better than those of traditional HA or TCP scaffolds with similar porosities. They also present non-cytotoxic and bioactive behavior, due to their carbon-based composition that also includes some residual mineral salts content.

## 1. Introduction

Tissue engineering and biomedical sciences have reached significant advances during the last years, which has led to a growing demand of new materials. Bone tissue engineering, and specifically bone grafting, has become a common surgical procedure all over the world, and the need of easily available and non-expensive materials is crucial to supply a proper medical attention and surgery for the worldwide population. Natural sources for bone grafts materials are highly limited, and the development of synthetic alternatives must be undertaken.

Synthetic bone scaffolds must comply with a compilation of lab requirements that qualify them as good candidates (or not) to be implanted into a living body [1]. Summarizing, the scaffold has to imitate as much as possible the physical and chemical characteristics of the natural bone, which is basically a mixture of some specific organic and inorganic compounds. Attending to its structure, a bone scaffold must combine a proper porosity to allow the cell adhesion, differentiation and proliferation and the vascularization, with good mechanical properties. Regarding to the chemical properties, it must present no cytotoxic behavior and should also have enough chemical affinity to promote the adhesion of cells. Finally, scaffolds generally require an appropriate degradation rate into living bodies.

The most commonly used materials for bone scaffolds are those already present in the natural bone composition, such as hydroxyapatite (HA) or similar calcium phosphates such as tricalcium phosphate (TCP) [2]. However, other families of materials are being currently developed to improve certain properties such as mechanic resistance or electrical conductivity [3]. Carbon-based materials such as carbon nanotubes [4], carbon dots [5], fullerenes [6], graphene oxide [7] or porous carbon monoliths [8] have been tested, and their potential has been demonstrated in bone tissue engineering, always as part of composite materials combined with HA, TCP, bioglasses or other bioactive materials.

Whey is the liquid resulting after casein precipitation in cheese production. Whey is mainly composed of water, sugars (lactose), proteins, fat and mineral salts [9]. It represents the largest fraction of the waste in dairy industries, amounting around 80 million tons per year all over the world [10]. Whey valorization mostly related to food industry and animal feeding due to its remaining nutritional content coping with almost 60% of the total waste generation [11]. The same nutrient rich composition makes whey a sensitive waste due to its huge Biological Oxygen Demand (BOD) and Chemical Oxygen Demand (COD) values [9]. As a part of the management process, liquid whey is generally converted into powders by spray-drying, obtaining a durable, storable and better transportable by-product known as whey powder.

Recently, whey has begun to be interesting as a new material for different applications. For example, whey protein isolate (WPI) has been found as a good precursor of aerogels with good mechanical properties that can be used as drug carriers [12]. WPI has also been studied combined with other materials such as bioglasses, calcium phosphates or other minerals to manufacture bone scaffolds [13,14]. Other authors suggest its possible application as an adhesive [15] due to its gluing properties. Furthermore, whey powders have been studied as a porous carbon precursor for energy storage [16]. Finally, porous carbon materials with excellent mechanical properties and virtually any shape have been also developed from whey powders [17].

In the present work, a porous carbon material obtained from whey powders is explored as an inexpensive, widely available and promising candidate for bone tissue engineering. This new porous carbon has very good mechanical properties, an acceptable porous structure, a non-cytotoxic chemical composition, biocompatibility and bioactive behavior.

## 2. Materials and Methods

### 2.1. Synthesis of Scaffolds

All the scaffold specimens were synthetized by casting, pouring whey powders into a mold with the desired shape. For this particular application, stainless steel molds were used, but ceramic or silicone molds are also adequate. Then, specimens were heated up to 150 °C for 1.5 h in absence of any external pressure, obtaining as a result a fragile but shaped monolith (green pieces) that can be demolded and handled. These green pieces were carbonized at different temperatures, 450 °C, 750 °C and 1000 °C, under inert atmosphere, using a N_2_ flow of 100 mL/min, a heating rate of 10 °C/min and a soaking time of 1.5 h, again in absence of any external pressure. The temperature of 450 °C was selected as the minimum temperature to obtain these whey-derived scaffolds since most of the volatiles are already removed at that temperature during the carbonization process of whey [17]. The monoliths were cooled down to room temperature under the same inert atmosphere. The final scaffolds were labelled as WDCS450, WDCS750 and WDCS1000 according to the respective carbonization temperatures.

**Figure 1:**
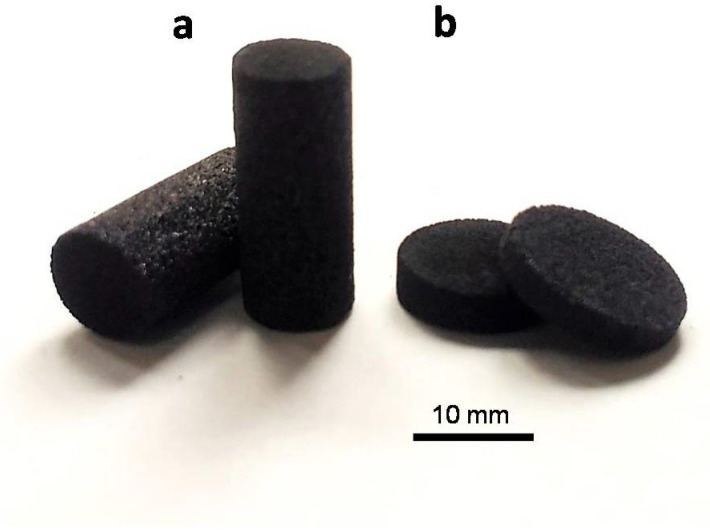
Specimens used in compressive strength tests (**a**); and in the assays of biological response, biodegradation and bioactivity (**b**). Scale bar, 10 mm.

For flexural and compressive strength assays, a degradation test and bioactivity assays in simulated body fluid (SBF), the monoliths were machined to obtain specimens with the proper shape required for each testing (see Figure 1). Thus, test specimens for bio-assays were discs of 10 mm of diameter and 2 mm high. For the compressive strength tests, specimens were shaped and sized as established in ASTM C1424-15 (see below) [18]. All pieces were lightly washed with distilled water after machining to remove any traces of dust or debris.

### 2.2. Characterization Techniques

Microstructural characterization and EDX analysis of the scaffolds were obtained by Field Emission Electron Microscopy (FESEM) using a Quanta FEG 650 (FEI, Eindhoven, Netherlands) equipment. Samples were placed on an aluminium tap using conductive double-sided adhesive tape.

Pore size distribution (PSD) was calculated from Hg intrusion carried out in a Micromeritics AutoPore IV porosimeter (Norcross, GA, USA) up to a maximum operating pressure of 227 MPa. The samples were previously outgassed by heating at 120 °C under vacuum over 12 h.

The real density of the materials (ρHe) was obtained using a Micromeritics AccuPyc 1330 pycnometer (Norcross, GA, USA), with He as the probe gas. Apparent densities (ρHg) were obtained from the Hg intrusion curves.

The intrinsic permeability of the whey-carbon scaffolds (k) was measured in a permeameter using He as flowing gas. The specimens were cylinders of 10 mm diameter and 10 mm length. Permeability was calculated using Darcy’s equation:

k = 2v_L_Lµ_gas_P_L_/(P_0_^2^ − P_L_^2^), (1)

where v_L_ is the permeameter cross section; L is the specimen length; µ_gas_ is the gas viscosity; P_L_ is the final pressure; and P_0_ is the initial pressure.

Compressive strength tests were carried out following the ASTM C1424-15 standard. The test consisted of applying a uniaxial compressive force to a cylindrical specimen until complete failure. A MTS Systems Corporation (Eden Prairie, MN, USA) model SMT1-100 N apparatus with a 5000 N cell was used. The nominal dimensions of the specimens were 6.35 mm of diameter and 12.70 mm length (Figure 1a). The final diameter value used for calculations was the average of three measurements on each specimen. Standard deviation of the diameter measurements was below 2% of the average value. The final value of the compressive strength and modulus of a given scaffold is the average of 15 tested specimens.

The formulas used for calculations are detailed below:

S_u_ = F_max_/A, (2)

E = Δσ/Δε,(3)

where S_u_ is the compressive strength (MPa); F_max_ is the maximum force (N); A is the cross-section (mm^2^); E is the modulus of elasticity (GPa); and Δσ/Δε is the slope of the engineering stress σ vs. the engineering strain ε curve within the elastic region.

Organic (CHNSO) elemental analysis was carried out in a LECO CHNS-932 and LECO VTF-900 microanalyzers (Vouersweg, The Netherlands). Elemental analysis of other inorganic elements was obtained by inductively coupled plasma mass spectrometry (ICP-MS) in an Agilent 7700 device (Santa Clara, CA, USA). Cl content was determined using an ion selective electrode (ISE) (Mettler-Toledo, Madrid, Spain). The point of zero charge (pH_pzc_) of the whey-carbon scaffolds was measured by grinding the pieces to a particle size below 0.212 mm and preparing a suspension of 250 mg of these powders into a certain volume of distilled water, keeping the suspension closed and continuously stirred, and adding water every 24 h. The pH_pzc_ value was obtained from the plateau of the pH variation curve measured daily [19].

The crystalline phases present on the scaffolds were characterized by X-ray diffraction (XRD, D8 Advance, Bruker, Hamburg, Germay), using Cu-Kα radiation (λ = 0.1542 nm) with an intensity of 40 mA and a current voltage of 40 kV. Diffraction profiles from 15° to 70° 2θ were obtained using a step size of 0.02° 2θ and a step time of 3 s.

### 2.3. Biocompatibility Test

#### 2.3.1. Cell Culture

Homo sapiens bone osteosarcoma cells (SaOs-2) were used for the in vitro test. The SaOS-2 cell line was kindly supplied by the Unit of Biotechnology and Biomedicine, Scientific Technical Services at the University of Oviedo (SCT-UNIOVI), Spain.

SaOs-2 human bone osteosarcoma cells were cultured at 37 °C in a 5% CO_2_ humidified atmosphere in McCoy’s 5A (Modified) Medium (Gibco, Thermo Fisher Scientific, Waltham, MA, USA) supplemented with 10% heat-inactivated Fetal Bovine Serum (Gibco, Thermo Fisher Scientific Inc., Waltham, MA, USA); 1% Penicillin-Streptomycin (10,000 U/mL) antibiotic solution (Gibco, Thermo Fisher Scientific, Waltham, MA, USA); 1% L-glutamine (2 mM) (Sigma Aldrich, Darmstadt, Germany) and 1% sodium pyruvate (110 mg/L) (Sigma Aldrich, Darmstadt, Germany). The cells were maintained at subconfluency and subcultured every 2–3 days. The cells were trypsinized using TrypLE Express reagent (Gibco, Life Technologies; Thermo Fisher Scientific Inc., Waltham, MA, USA).

#### 2.3.2. In Vitro Cytotoxicity Indirect Assays

In vitro cytotoxicity was evaluated according to ISO 10993-5 standards [20]. These methods specify the incubation of cultured cells in contact with a device and/or extracts of a device either directly or through.

Extraction samples were sterilized at 121 °C over 20 min; then, 1 mL of McCoy’s 5A supplemented with 5% Newborn Bovine Calf Serum (NBCS, HyClone, GE Healthcare Life Sciences, Madrid, Spain) and 1% Penicillin-Streptomycin (10,000 U/mL) antibiotic solution (1 vol%) (Gibco, Thermo Fisher Scientific, Waltham, MA, USA) were added to each sample and incubated at 37 °C in a 5% CO_2_ humidified atmosphere for 24 h. Cell culture medium alone was incubated under identical conditions to serve as a negative control extract.

In the assay procedure, 96 well plates were seeded with 2.5 × 10^4^ SaOs cells/well in 100 μL McCoy’s 5A medium supplemented with 5% Newborn Bovine Calf Serum (NBCS, HyClone, GE Healthcare Life Sciences, Madrid, Spain) and 1% Penicillin-Streptomycin (10,000 U/mL) antibiotic solution (1 vol%) (Gibco, Thermo Fisher Scientific, Waltham, MA, USA) and incubated under cell culture conditions for 24 h. Thereafter, the cell culture medium was discarded, and 100 μL of extract were added to each well. Cells were further incubated for 24 h and then subjected to the Neutral Red uptake assay.

The medium was removed, and the cells were washed twice with phosphate-buffered saline (PBS). A total of 100 µL of Neutral Red (NR, Scharlau, Scharlab S.L., Barcelona, Spain) solution (95% NBCS medium and 5% NR dilution, 0.4 wt% NR in distilled H_2_O) were added to each well and incubated for 3 h to react with the cells. After 3 h, the NR solutions were removed, and the plates were washed twice with PBS; then, 150 µL of desorption reagent (50% ethanol absolute partially denatured technical grade, Panreac Química S.A., Madrid, Spain; 49% distilled H_2_O and 1% glacial acetic acid, Panreac Química S.A., Madrid, Spain) were added to each well. After adding the desorption reagent, absorbance at a wavelength of 540 nm (OD_540_), which is directly proportional to the number of living cells in culture, was measured using a BIO-RAD Model 680 Microplate Reader (Bio-Rad, Hercules, CA, USA). All assays were performed in triplicate.

Three controls were used in this experiment: a positive control, wells containing cells and NBCS culture medium with 2% Triton X-100 (Sigma-Aldrich, Darmstadt, Germany); a negative control, wells containing cells and NBCS culture medium; and a blank control, empty wells containing NBCS culture medium.

Thus, the percentage viability was calculated according to the equation:

% viability = 100 × (OD_540_ samples/OD_540_ negative control) (4)

### 2.4. Degradation and Bioactivity

A biodegradation test of porous scaffold was carried out by immersing 0.06 g of scaffold (Figure 1b) in 10 mL of buffer solution (Tris-HCl). The buffer solution was prepared according to EN ISO 10993-14:2001. The Tris-HCl solution was prepared by dissolving tris-hydroxymethylaminomethane (ACS reagent, ≥99.8%, Sigma-Aldrich, Darmstadt, Germany) in water (grade 2) with buffering at pH 7.4 ± 0.1 by 1 mol/L hydrochloric acid (ACS reagent, 37%, Sigma-Aldrich, Darmstadt, Germany) at 37 °C. Samples were extracted from the buffer after given times of 6, 24, 72, 168, 336 and 672 h.

The assessment of the scaffold bioactivity was carried out using the standard in vitro protocol developed by Kokubo and Takadama Kokubo [21]. In fact, in order to mimic in vitro the possible formation of hydroxyapatite onto the scaffolds, the samples were immersed in flasks containing 10 mL of SBF and maintained at a controlled temperature of 37 °C. Samples were extracted from the SBF after given times of 6, 24, 72, 168, 336 and 672 h. A periodic refresh (every 48 h) of the SBF was used to simulate the circulation in the human body.

Once extracted from the buffer solution or SBF, the samples were rinsed with acetone and left to dry at 100 °C for 24 h. The concentration of ions was measured by ICP-MS (7700x, Agilent, Santa Clara, CA, USA) spectrometry. Triplicate samples were analyzed for each immersion time and averaged results calculated.

## 3. Results

### 3.1. Morpho-Structural Characterization of the Whey-Derived Carbon Scaffolds

**Figure 2:**
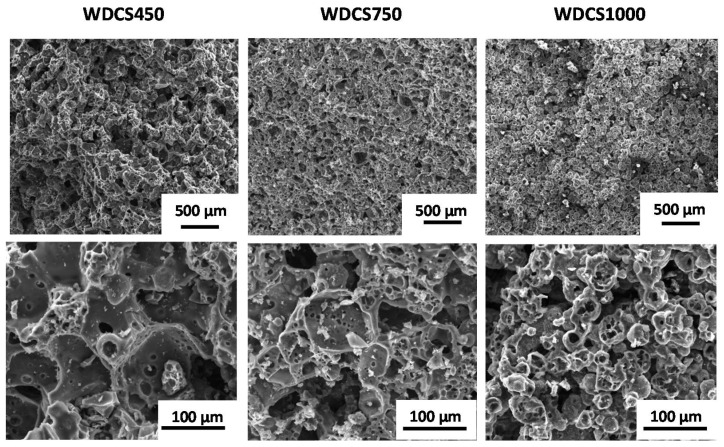
FESEM micrographs of scaffolds obtained from the sintering of whey powder at 450 °C, 750 °C and 1000 °C. Scale bars: 500 µm (top images); 100 µm (bottom images).

FESEM micrographs of whey-derived carbon scaffolds (WDCS) prepared at different carbonization temperatures are shown in Figure 2. It is noticeable how carbonized powders particles are stuck to each other through a sintering-like process. This fact is explained on the basis of the Maillard Reaction, a group of reactions between sugars and proteins favored by heating, combined with the gelation of whey proteins [17]. Once the particles are stuck together, whey powders conform into a monolith shaped by the mold used. Then, as temperature rises, the monolith carbonizes without shape modification, apart from the shrinkage, giving rise to the WDCSs.

All the scaffolds obtained, no matter the temperature of carbonization, have a similar structure consisting in a heterogeneous interconnected porous network of different pore dimensions up to 300 µm maximum. The carbonized monoliths experiment demonstrated a linear shrinkage, hence a volume reduction as carbonization temperature rises, so the pore network reaches the maximum pore sizes at lower temperatures, being 248.8 µm for 450 °C, 162.8 µm for 750 °C and 162.8 µm for 1000 °C, according to FESEM analysis. This was further confirmed by Hg intrusion (see Table 1). Hg intrusion points out that the materials present also contributes to the mesopore (pore size <100 nm) range. The predominant pore sizes are 33 µm, 28 µm and 25 µm for WDCS450, WDCS750 and WDCS1000, respectively. Furthermore, scaffolds become denser at higher temperatures. Typical values of the linear shrinkage of the carbon scaffolds ranged from 16% at 450 °C to 22% at 1000 °C.

### 3.2. Mechanical Characterization of the Whey-Derived Carbon Scaffolds

**Figure 3:**
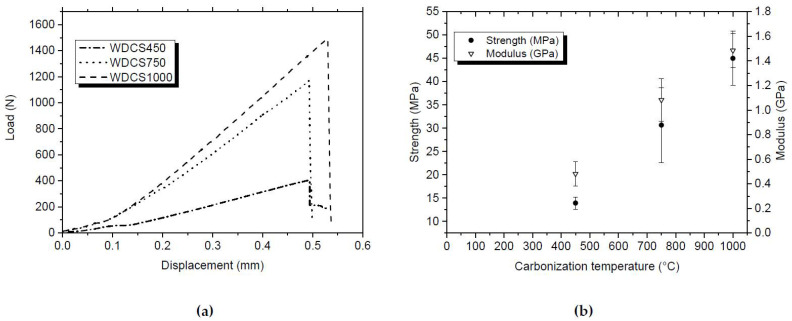
(**a**) Selected examples of the compressive tests carried out with WDCSs; (**b**) compressive strength and modulus of the WDCSs prepared at different carbonization temperatures.

Compressive tests of the WDCSs show typical elastic stress-strain curves (see Figure 3a). The highest compressive strength corresponds to scaffolds carbonized at higher temperatures, reaching the maximum of 45 MPa in the case of WDCS1000, and decreasing to 30 MPa and 13 MPa for WDCS750 and WDCS450, respectively. The compressive stiffness of the whey carbons also increases with temperature, resulting in compressive elastic moduli of 1.5 GPa, 1.1 GPa and 0.5 GPa, for carbonization temperatures of 1000 °C, 750 °C and 450 °C, respectively (Figure 3b). The errors bars of the WDCS750 sample in Figure 3b are relatively high due to the occurrence of defects on the test specimens after their machining. Since the nominal dimensions of all test samples are the same, results shown in Figure 3a indicate that the (calculated) strain at failure would be very similar for all materials.

### 3.3. Chemical Analysis

Composition of whey powders is shown elsewhere [17]. These are basically a combination of 74.8 wt% of sugars (lactose) and 12.7 wt% of proteins (β-lactoglobuline), which contribute to implement Maillard reactions when heated [22]. The remaining part contains fats (1.8 wt%), moisture (2.7 wt%) and inorganic matter (4.4 wt%).

Regarding its inorganic matter (Table 2), the presence of calcium (0.4 wt%) and phosphorous (0.6 wt%) is remarkable, since they are the species of interest from the bone growing perspective. These species, in the form of calcium phosphates, are naturally present in milk and represent the 80 wt% of total calcium content [23]. In addition, they are used as an additive to improve cheese and other dairy products properties such as texture or fat emulsion [24]. The remaining percentage (up to 100%) of the whey powder composition is normally ascribed to trace elements, mainly Fe and Mn.

Maillard reactions help to consolidate the structure of monoliths before carbonization, which makes it possible to obtain final consistent and shaped whey-carbon scaffolds [17]. These final scaffolds are mostly carbon, but there are also other elements present in the form of ashes. These ashes are most likely responsible of the basic pH_pzc_ of all WDCSs with values ranging from 10 to 11 pH units. Elemental analyses of the scaffolds are shown in Table 3.

**Figure 4:**
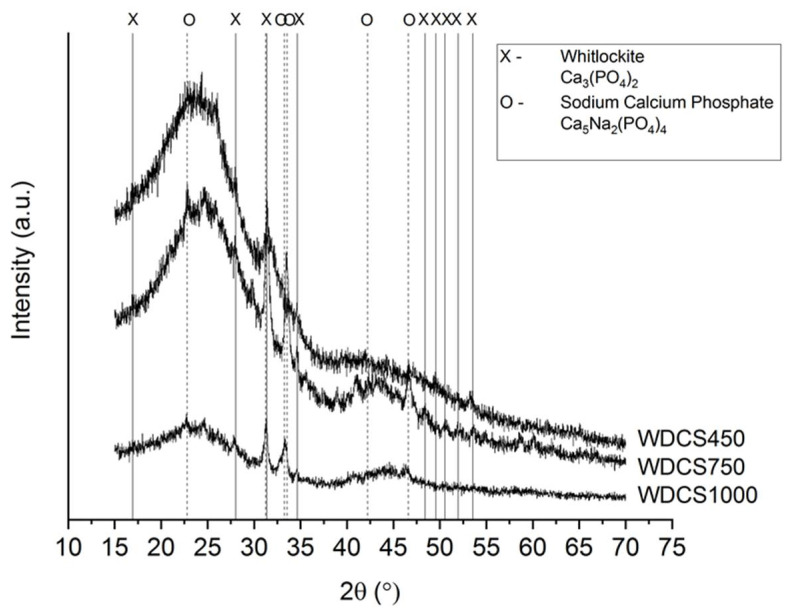
XRD diffractograms of the whey-carbon scaffolds.

A high content of inorganic matter is generally undesirable for some porous carbon applications. However, to be used as scaffolds in bone regeneration, the presence of calcium phosphates favors the bioactivity, cellular function and expression and osteoconductivity of the scaffolds [25]. Two types of calcium phosphates, whitlockite and sodium calcium phosphate, are the main crystalline species detected in the WDCSs (Figure 4).

### 3.4. Biological Response

**Figure 5:**
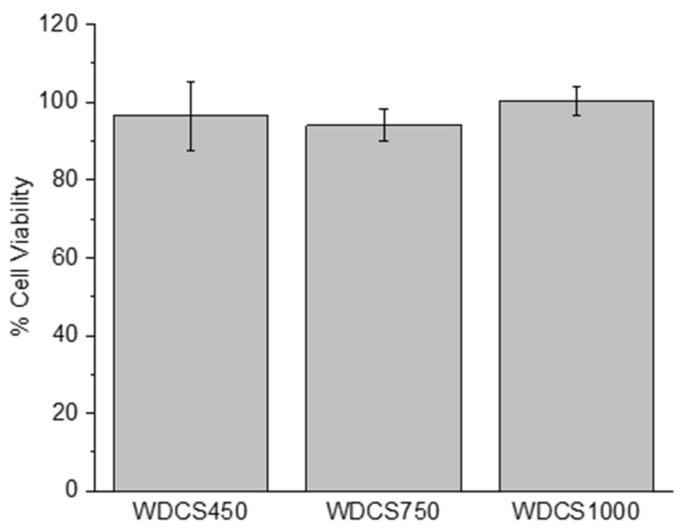
Cell viability percentage of SaOs-2 cells after incubation during 24 h with extracts of WDCS450, WDCS750 and WDCS1000.

A material is considered non-cytotoxic when it allows for over 70% cell viability, as specified in ISO 10993-5. Cytotoxic effects were analyzed by quantifying the number of living cells in the media after culturing SaOs-2 cells for 24 h in contact with extracts of the samples. As it is shown in Figure 5, none of the tested structures induced sufficient cell death to be considered cytotoxic.

### 3.5. Biodegradation and Bioactivity Results

**Figure 6:**
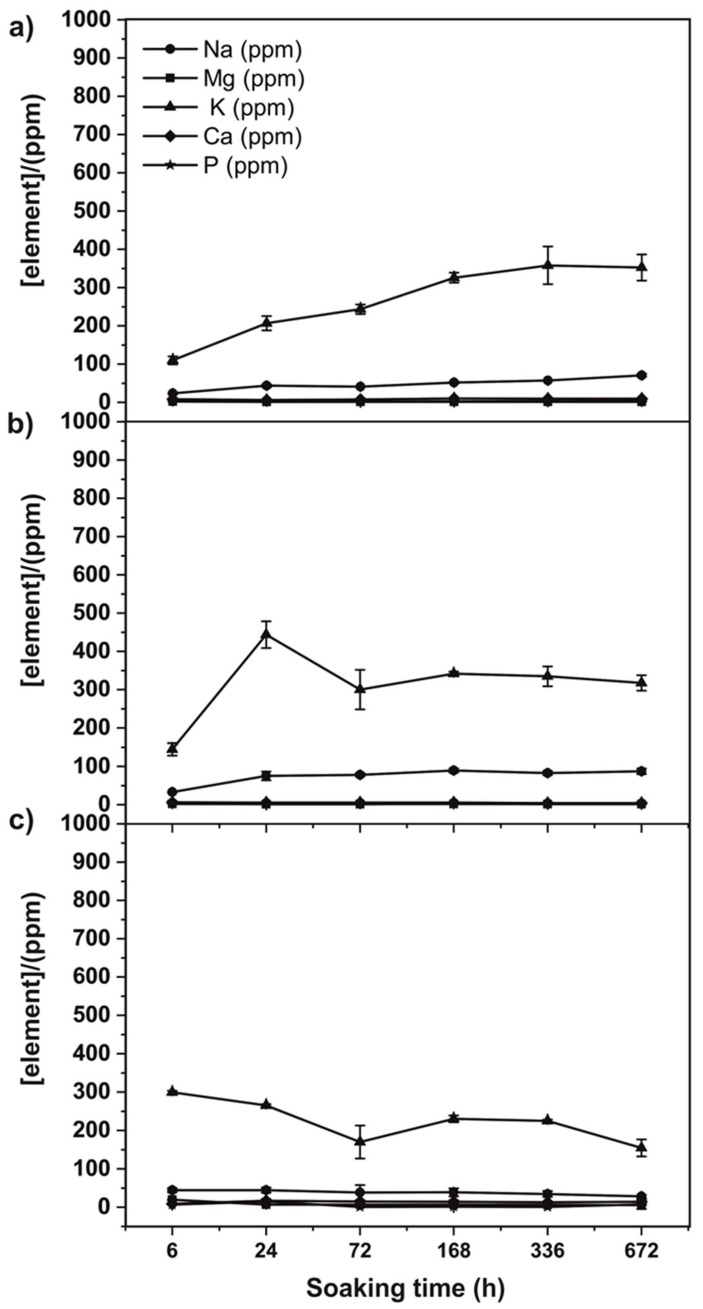
Biodegradation behavior of the WCDSs: Changes in the ions concentration vs. soaking time in (**a**) WDCS450, (**b**) WDCS750 and (**c**) WDCS1000.

The 3D scaffolds were immersed in Tris-HCl buffer for different intervals of time in order to determine the scaffold bioresorption. During the immersion in Tris-HCl solution, the scaffolds’ composition was altered, and some inorganic elements were leached to the buffer solution, as determined by ICP-MS. Figure 6 shows an increase in the concentration of Na^+^ and K^+^ with time. Leaching of those particular ions was more evident in the case of the WCDS450, with the concentration of Na^+^ and K^+^ increasing as the soaking time increases. In the case of WDCS750, this lixiviation was observed in the first stages of the test, and then it stabilized. The lixiviation of ions in WDCS1000 is very small compared to the samples treated at lower temperatures.

**Figure 7:**
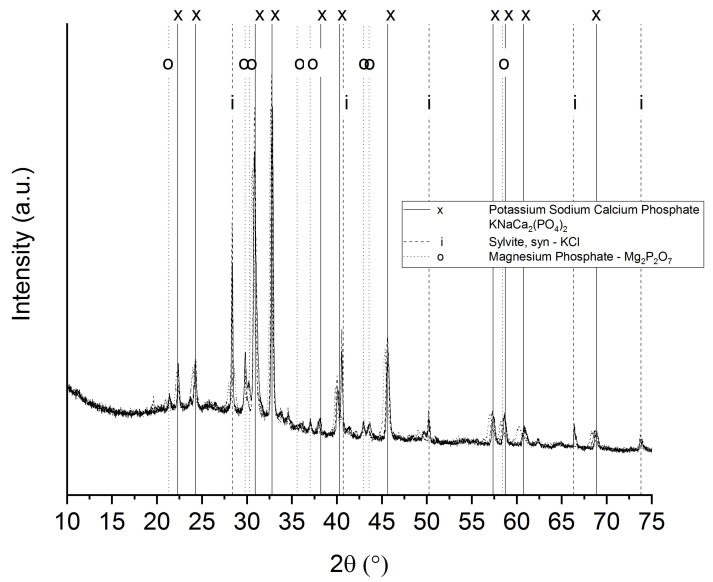
XRD diffractograms of the ashes of carbonized whey scaffolds at 450, 750 and 1000 °C (overlapped).

In all cases, the leaching of K^+^ and Na^+^ is related to the ash content of the scaffolds. The XRD of those ashes obtained after burning off the scaffolds is shown in Figure 7. All samples present the same main crystalline moieties: sylvite (KCl), magnesium phosphate and a potassium sodium calcium phosphate.

**Figure 8:**
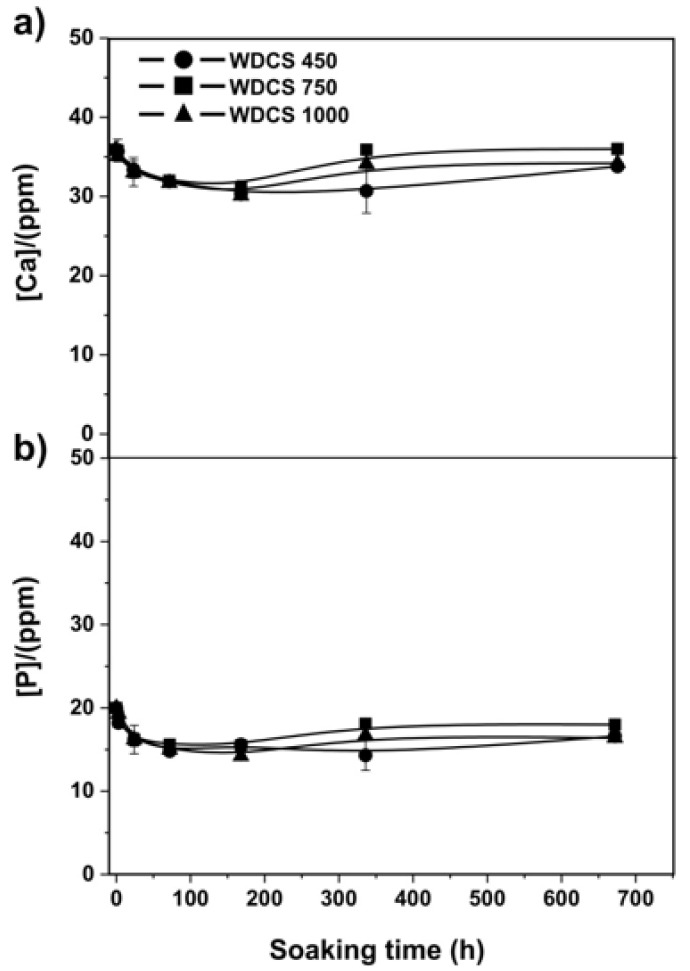
Bioactivity behavior of the carbon scaffolds: Changes of the Ca (**a**) and P (**b**) concentrations vs. soaking time in WDCS450 (Circle), WDCS750 (square) and WDCS1000 (triangle).

The scaffolds were incubated in SBF over 672 h to study their bioactivity. In this case, the concentration of Ca and P was monitored with soaking time (Figure 8). Results show that the concentration of calcium and phosphorus decreases in the first stages of the assay (100–200 h), suggesting that calcium and phosphate ions could form a hydroxycarbonate apatite (HCA) phase on the scaffold surface due to a process of nucleation and precipitation.

From the ICP-MS results, the percentage of calcium and phosphorus in the SBF was calculated. Results are collected in Table 4. A higher percentage of ions in the SBF means less deposition of them on the scaffold surface. In this sense, the lower content of Ca and P ions was observed for the scaffold treated at 750 °C, indicating the higher bioactivity of this sample when compared to the scaffolds treated at 450 °C and 1000 °C.

**Figure 9:**
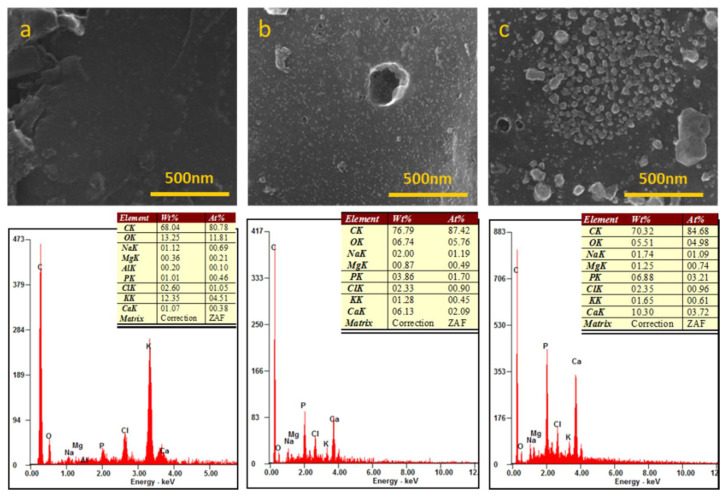
FESEM micrographs and EDX analyses of WDCS750 (**a**) before SBF assay, (**b**) after 1 week in SBF and (**c**) after 4 weeks in SBF. Scale bars (top images): 500 nm.

Figure 9 shows FESEM microstructures of the scaffold treated at 750 °C and incubated in SBF after 1 and 4 weeks. The white inclusions on the surface of the scaffold correspond to the deposition of calcium phosphates compounds on the surface of the scaffold. According to the EDX analysis, the content of Ca and P on the scaffold increases with time with respect to the reference sample, i.e., WDCS750 before the analysis.

## 4. Discussion

Porosity is a key factor of the materials envisaged for bone regeneration. Osteoblasts have a size between 10 µm and 50 µm, and osteogenic response takes place in pores >50 µm, although, according to other authors, optimal pore size for this purpose is comprised between 100 µm and 500 µm [26]. Larger pores do not contribute to improve cell adhesion [26]. On the other hand, micropores (pore size <10 µm) increase the surface area and present a positive influence on ion interchange and bone protein adsorption [27].

Regarding the pore morphology, all WDCSs present a structure of empty spheres fused to each other (Figure 2). This fact results in an abundance of curved and concave surfaces, which is highlighted by several authors as an advantage for tissue development since it looks more as natural cancellous bone morphology [27]. Furthermore, the heterogeneous particles and pore sizes in WDCSs result in rough surfaces, which is positive for union, differentiation and proliferation of osteogenic cells [28].

WDCSs present an acceptable open porosity percentage, between 48 and 58%, which are considered optimum values by some authors [29]. The materials also have a proper pore size distribution. However, the abundance of micropores and mesopores has a negative effect on the permeability and pore interconnection, which is a very important factor to consider from the perspective of cell and fluid migration. It should be pointed out that the synthesis of these scaffolds was made without any pore size control method. The great versatility of this material and the easiness of handling, molding and machining gives a great deal of room for improvement in this regard.

The best sample from the point of view of permeability is WDCS750 rather than WDCS450, i.e., despite the shrinkage of the WDCSs during the carbonization, pointing out that the higher the temperature is, the smaller the pore size and so the permeability. This apparent contradiction is explained by the significant increase in the real density (ρHe) of the scaffolds when the carbonization temperature rises from 450 °C to 750 °C (Table 1). As a consequence, the open porosity of WDCS750 is significantly higher than that of WDCS450 (Table 1). Therefore, WDCS750 seems the best sample from the point of view of morphological requests, combining a proper pore size, optimal open porosity percentage and the best permeability of all WDCSs. However, whey-carbon permeability is lower than those of other reference scaffold materials such as hydroxyapatite, which presents permeability values about 7.5 × 10^−11^ m^2^ for scaffolds with similar porosities than those of the WDCSs [30]. Moreover, the trabecular bone has a much lower permeability, with k between 1–11 × 10^−9^ m^2^ [31].

On the other hand, the results of compressive strength indicate that the carbonized whey monoliths are exceptional materials, especially when considering their high porosity values. Table 5 collects a comparison between the main structural properties of whey-carbon scaffolds, as well as of other scaffolds and human cancellous bone. The compressive strength of the whey-carbon scaffolds clearly stands out.

A scaffold is defined as a porous matrix developed to provide an appropriate microenvironment that promotes tissue repair and regeneration, since it serves as a temporary structure that is replaced by native tissue subsequently, i.e., it needs to be gradually removed. WDCSs are carbon-based materials, so they were expected to be mostly persistent within the Tris-HCl solution. Some degradation is nonetheless observed, corresponding to the lixiviation of chemical species that conform the inorganic matter (ashes) of the scaffolds (Figure 7). According to Figure 6, Na^+^ and K^+^ ions are fundamentally lixiviated from the scaffold surface, this effect being more evident in the case of the sample treated at 450 °C (Figure 6a), where the concentration of those ions increases as the soaking time increases. In the case of samples carbonized at 750 °C (Figure 6b) and 1000 °C (Figure 6c), leaching happens at the beginning of the assay, and then stabilization of the ions concentration is observed. This result could be attributed to the pore size of the carbonized samples. It is known that porosities and pore sizes influence the degradation rates and scaffolds with a larger pore size degrading more rapidly [42]. In this way, a sample sintered at 450 °C shows the higher pore size (see Figure 2) and the higher degradation rate.

It is thus clear that WDCSs show only a limited ionic degradation. The lack of a significant degradation of the carbon structure shown by the in vitro tests could not be determined, as far as it has been described how other carbon-based materials can be degraded by the action of oxidative enzymes when they are inside the human body [43]. Moreover, the ionic degradation of scaffolds has been highlighted to play an important role in their osteogenesis and angiogenesis process [44]. In the case of WDCS samples, the eventual release of ions does not comprise the non-cytotoxicity of the materials, as shown in Figure 5.

In vitro bioactivity tests evaluate the ability of the formation of HA or carbonate HA on the surface of the biomaterials, after soaking in SBF. The results show that the concentration of calcium and phosphorus decreases in the first stage of the assay. In the case of the scaffold treated at 750 °C, white inclusions of calcium phosphate crystals can be observed on the scaffold surface after 1 and 4 weeks of soaking time (Figure 9). According to the literature [45], the process of HA deposition happens in three steps: (1) the interaction of the surface of the scaffold with the calcium ions present in the SBF forming a carbonated hydroxyapatite, (2) the interaction with the phosphate ions present in the solution and (3) crystallization of HA on the surface of the scaffold. It is speculated that this HA deposition process is favored by the presence of crystalline calcium phosphates on the surface of the WDCSs [25].

## 5. Conclusions

Whey powders can be used as precursors to obtain porous carbon materials with suitable properties for bone tissue engineering. Powder particles stick together in a pseudo-sintering process promoted by heating. The resulting materials present an open porosity comprised between 40 and 60%, consisting of a hierarchical pore network with pore sizes from a few nanometers up to 300 microns. Whey-derived carbon scaffolds also present excellent mechanical properties, especially when compared to the compressive strength of other materials with equivalent porosity values used frequently as bone scaffolds. Whey-derived carbon scaffolds are non-cytotoxic due to their chemical composition, mostly C, and their residual content in mineral salts of Ca and P generates enough affinity to promote an incipient bioactive behavior.

## Figures and Tables

**Table 1:** Densities, porosity, permeability, predominant, maximum and minimum pore sizes and linear shrinkage of the WDCSs.

	ρ_Hg_^a^	ρ_He_^c^	Porosity ^d^	k ^f^	d_p_^a,b^	Max d_p_^a^	Min d_p_^a^	Shrinkage
	(g/cm^3^)	(g/cm^3^)	(%)	(m^2^)	(µm)	(µm)	(µm)	(%)
WDCS450	0.78	1.61	52	8.76 × 10^−13^	33.3	248.8	5 × 10^−3^	15.9 ± 0.7
WDCS750	0.80	1.91	58	1.46 × 10^−12^	27.7	162.8	5 × 10^−3^	22.9 ± 0.3
WDCS1000	1.06	2.04	48	8.90 × 10^−13^	25.3	162.8	5 × 10^−3^	21.9 ± 2.4

^a^ Hg intrusion; ^b^ maxima of the Hg PSD; ^c^ He intrusion; ^d^ Porosity = [1 − (ρHg/ρHe)] × 100; ^f^ He permeability coefficient. Cylinders of diameter = 1 mm and length = 25 mm were synthetized to carry out the permeability experiments.

**Table 2:** Elemental analysis of the whey powders.

Element ^1^ (wt%)	
C ^2^	40.7
H ^2^	6.3
N ^2^	2.3
O ^2^	46.1
S ^2^	0.2
Na ^3^	0.3
K ^3^	1.2
Mg ^3^	0.1
Ca ^3^	0.4
P ^3^	0.6
Cl ^4^	0.2

^1^ Dry basis; ^2^ Organic (CHNSO) elemental analysis; ^3^ ICP-MS; ^4^ Chloride ISE.

**Table 3:** Elemental analysis and ash content of the WDCSs obtained at different carbonization temperatures.

(wt%) ^a^	WDCS450	WDCS750	WDCS1000
C	70.9	74.3	76.1
H	3.2	1.1	0.6
N	3.6	3.0	2.0
O	14	13.6	11.2
S	0.2	0.1	0.1
Ash	11.3	13.6	13.7

^a^ Dry basis.

**Table 4:** Percentage of Calcium and Phosphorus within the SBF.

SAMPLE	% Calcium	% Phosphorus
WDCS450	15.6	28.5
WDCS750	13.4	22.5
WDCS1000	16.1	29

**Table 5:** Comparison between different bone scaffolds with similar porosity.

Scaffold Composition	Porosity	Pore Size	Compressive Strength	Permeability	Ref
	(%)	(µm)	(MPa)	(m^2^)	
WDCS750	58	1–200	30	1.46 × 10^−12^	This work
HA	50	250	-	7.5 × 10^−11^	[31]
HA	60	50	12	-	[32]
HA/TCP	60	100–200	5	-	[33,34,35]
WPI/aragonite	15.7	18–369	3.16	-	[14]
Cancellous bone	30–90	100–600	0.70–15	0.12–8 × 10^−11^	[34,36,37,38,39,40,41]

## Data Availability

Data are contained within the article.
